# Exploring the causal relationship between female reproductive traits and asthma: A bidirectional two-sample Mendelian randomization study in a European population

**DOI:** 10.1097/MD.0000000000044720

**Published:** 2025-09-26

**Authors:** Dan Sun, Xingjia Wang, Xinyu Han

**Affiliations:** a Youth League Committee, Heilongjiang University of Chinese Medicine, Harbin, China; b Department of First Clinical Medical College, Heilongjiang University of Chinese Medicine, Harbin, China; c Department of Gynecology, Wenzhou Traditional Chinese Medicine Hospital Affiliated to Zhejiang Chinese Medical University, Wenzhou, China.

**Keywords:** asthma, causality, Mendelian randomization, women’s reproductive traits

## Abstract

Epidemiological studies have reported gender disparities in asthma, suggesting female reproductive traits may play a role, though causality remains unclear. We used summary data from genome-wide association studies on 12 female reproductive traits and asthma (408,422 European individuals). Univariate (UVMR) and multivariate Mendelian randomization (MVMR) analyses were performed using inverse-variance weighted (IVW) as the primary method. Supplementary methods included MR-Egger, weighted median, and robust adjusted profile score for UVMR, and MVMR-Lasso and MVMR-median for MVMR. Sensitivity analyses were conducted to validate findings. We acknowledge that MR estimates rely on key assumptions (relevance, independence, exclusion restriction), and violations may bias causal inference. After Bonferroni correction, UVMR-IVW showed that later age at first sexual intercourse (AFS) significantly reduced asthma risk (OR = 0.663, 95% CI = 0.603–0.729, *P* < .001). Later age at first birth (AFB) (OR = 0.893, 95% CI = 0.862–0.926, *P* < .001) and last birth (ALB) (OR = 0.845, 95% CI = 0.755–0.946, *P* < .001) were also protective. Age at menarche (AAM) showed a nominally significant inverse association (OR = 0.968, 95% CI = 0.94–0.998, *P* = .034), while hormone replacement therapy (HRT) use increased asthma risk (OR = 1.435, 95% CI = 1.05–1.962, *P* = .024). No causal links were found for age at natural menopause, live births, stillbirths, miscarriages, hysterectomy, oophorectomy, or oral contraceptive use. In MVMR analyses adjusted for body mass index (BMI), educational attainment, smoking, and Townsend deprivation index, the protective effects of AFS (OR = 0.678, 95% CI = 0.552–0.833, *P* < .001) and AFB (OR = 0.933, 95% CI = 0.874–0.995, *P* = .033) persisted. Mediation analysis indicated BMI partially mediated the AFS and AFB-asthma associations. This MR study provides evidence consistent with a causal effect of earlier AFS and AFB on increased asthma risk, partially mediated by BMI. These findings underscore the potential importance of reproductive and metabolic health in asthma prevention strategies.

## 1. Introduction

Asthma, a prevalent and heterogeneous condition found in both children and adults, is characterized by reversible restrictions in expiratory airflow, accompanied by diverse respiratory symptoms. Globally, there are approximately 358 million individuals living with asthma, representing a significant medical burden on society.^[1]^ Increasing evidence suggests notable gender differences in asthma. In the United States, statistics indicate that prepubescent boys have a 30% to 50% higher incidence of asthma compared to girls. However, this trend reverses in adulthood, with prevalence rates of 5.5% in adult males and 9.7% in adult females.^[2]^ Moreover, adult women are at a greater risk of developing severe asthma, exhibiting higher rates of hospitalization and mortality.^[3]^ The distinct reproductive traits of women and the hormonal fluctuations associated with these characteristics might account for the gender differences observed in asthma. However, the existence of a causal link between female reproductive factors and the risk of asthma remains uncertain. Throughout their lifespan, women experience a range of reproductive characteristics including Age at menarche (AAM), age at first sexual intercourse (AFS), age at first birth (AFB), age at last birth (ALB), the number of live births, the number of abortions, and age at natural menopause (ANM). Additionally, reproductive-related behaviors such as hysterectomy or oophorectomy, the use of oral contraceptive use (OC), and hormone replacement therapy (HRT) can lead to significant hormonal fluctuations in women. The multiple reproductive factors mentioned have been identified as risk factors for asthma. This association may be attributed to the effects of changes in sex hormones on cells related to immune inflammation in the airways.^[4]^

Previous meta-analysis has demonstrated that girls who experience early menarche (≤ 12 years old) face an increased risk of asthma compared to those with later menarche (odds ratio [OR] = 1.37, CI = 1.15–1.64, *P* = .0005).^[5]^ Conversely, other cohort studies have reported conflicting results. Notably, 2 of these studies found no significant association between AAM and the risk of developing adult asthma.^[6,7]^ As socioeconomic conditions evolve, there’s a noticeable trend of delayed pregnancy among women of reproductive age, with the average age of first childbirth reaching approximately 30 years in many countries.^[8]^ A cohort study from Taiwan has demonstrated that an increase in the age at first childbirth correlates with a heightened risk of death due to asthma. Concurrently, it was observed that the adjusted risk ratio for asthma death significantly decreases as the number of live births increases.^[9]^ Furthermore, a recent prospective cohort study has identified a significant correlation between various reproductive factors and the risk of adult-onset asthma in women. The study’s findings indicate that an early age at the first live birth, frequent miscarriages (two or more) or stillbirths (two or more), surgically induced menopause (including hysterectomy or oophorectomy), and the use of HRT are all linked to an increased risk of developing asthma in adulthood.^[10]^

The evidence linking reproductive factors to asthma, primarily derived from observational studies, is subject to inherent limitations in addressing confounding factors and reverse causality bias. Conventional epidemiological studies are often confounded by socioeconomic status, health behaviors, and unmeasured variables, and cannot easily distinguish whether reproductive traits influence asthma risk, or whether asthma-related factors (e.g., chronic inflammation or corticosteroid use) influence reproductive timing or choices. To surmount these challenges, Mendelian randomization (MR) has been proposed as a more robust approach. MR utilizes genetic variations closely associated with the exposure of interest as instrumental variables (IVs) to evaluate the impact of this exposure on outcomes.^[11]^ This method is akin to randomized controlled trials in its conceptual framework. The random allocation of genetic variations during meiosis means that MR findings are less prone to the influences of confounding variables and reverse causal relationships, thereby providing a more reliable analysis of causal connections.^[12,13]^ MVMR is an advanced approach that incorporates genetic variations of multiple risk factors into a cohesive model. This method allows for the simultaneous evaluation of various exposures while effectively minimizing the impact of confounding factors.^[14]^ Furthermore, considering that the relationship between reproductive traits and asthma may be bidirectional – for example, asthma-related systemic inflammation, HPA axis disruption, or long-term medication use might influence reproductive timing or outcomes – we conducted bidirectional MR analyses to explore both directions of potential causal effects. Consequently, we carried out both UVMR and MVMR studies to ascertain the bidirectional causal relationships between multiple reproductive behaviors and asthma.

## 2. Materials and methods

### 2.1. Study design

**Figure 1.**
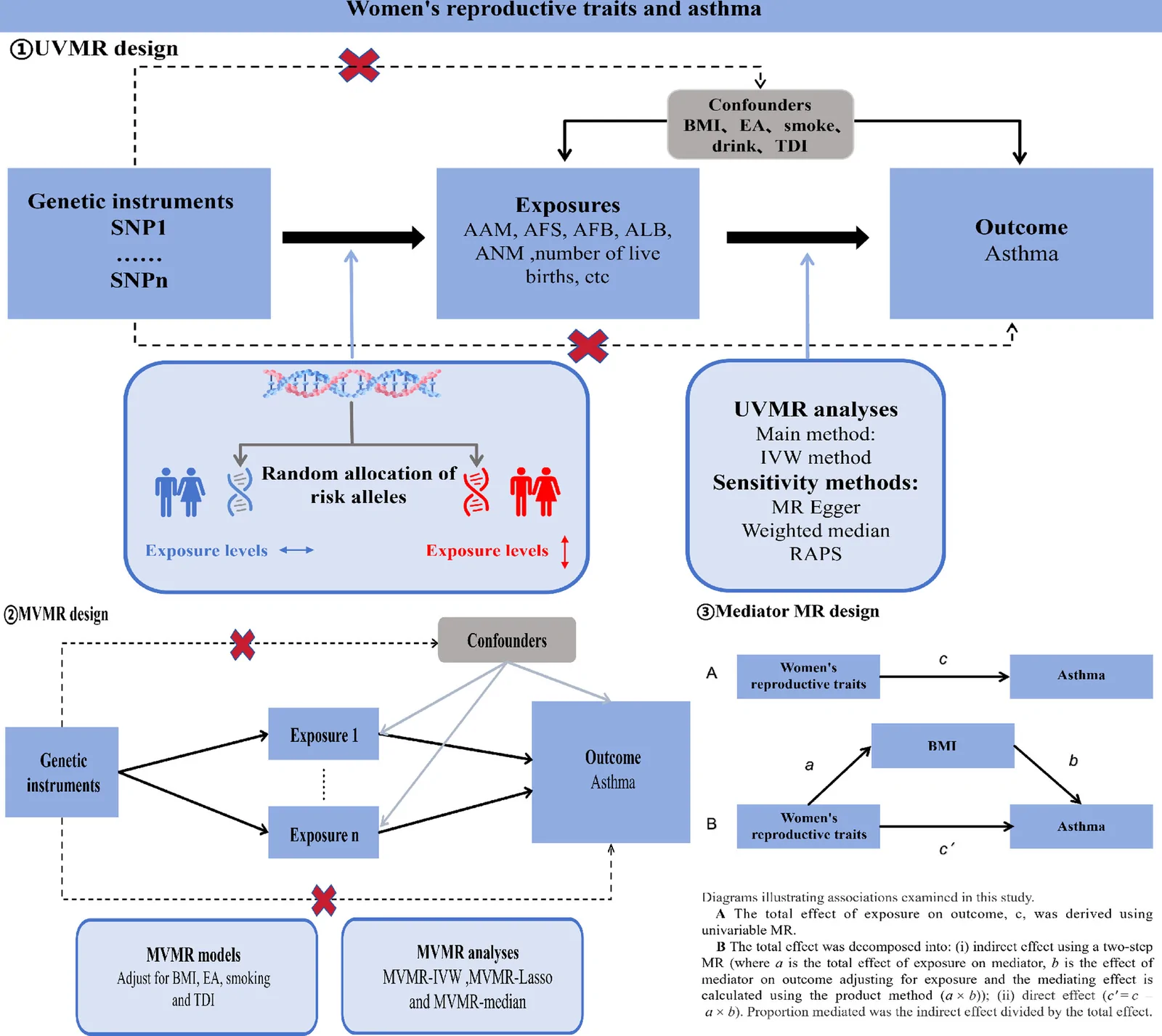
Assumptions and study design of the MR study of the associations between 12 women’s reproductive traits and asthma.

A concise depiction of the two-sample MR designs was presented in Figure 1. We conducted two-sample UVMR and MVMR analyses to thoroughly investigate the associations of 12 women’s reproductive traits with asthma. UVMR relies on 3 primary assumptions: the genetic variants chosen as the IVs should be strongly linked to the exposure; the genetic variants should not be correlated with confounding factors; the genetic variants influence the outcome solely through the exposure and not via alternative pathways.^[15]^ The primary premise of MVMR, in contrast to UVMR, pertains to genetic variation related to multiple exposures, while the remaining assumptions align with UVMR.^[16]^ Initially, genetic variables linked to each woman’s reproductive traits were selected to infer the causal relationship with asthma utilizing UVMR. Prior observational clinical trials and MR investigations have presented evidence indicating that body mass index (BMI), educational attainment (EA), smoking, and TDI are risk factors for asthma development.^[17–20]^Consequently, the genetic variations relevant to these 4 exposures were incorporated into the MVMR model for the purpose to estimate the direct impact of reproductive factors on asthma.

### 2.2. Data sources

In the present study, the exposures were reproductive characteristics of women, including AAM, ANM, AFS, AFB, ALB, number of live births, number of stillbirths, number of SM, history of hysterectomy or bilateral oophorectomy, ever taken OC pill, and ever used HRT. The research outcome under investigation was asthma. The summary statistical data for AAM originates from the genome-wide association studies (GWAS) conducted by Loh et al, with a sample size of 279,470 and a total of 11,971,701 single-nucleotide polymorphisms (SNPs) identified.^[21]^ The genetic IVs for AFS and AFB were acquired from the largest GWAS meta-analysis of 36 European ancestry studies, as reported by Mills et al. This meta-analysis encompassed 397,338 and 542,901 females, respectively. AFS and AFB were both treated as continuous measures. For AFS, individuals who provided invalid answers or reported ages below 12 years were excluded, and the data were then transformed using inverse rank-normalization. AFB was assessed specifically for individuals who had experienced childbirth. The genetic relationships between SNPs and AFB or AFS were corrected for the respondent’s birth year, as well as its square, cubic, and top principal components.^[22]^ The database of remaining 9 phenotypes related to female reproduction, including ANM, number of live births, and number of stillbirths etc, were derived from Ben Elsworth recently published GWAS study, involving a total of 9851,867 SNPs. “ https://gwas.mrcieu.ac.uk/datasets/ ”(accessed on March 17, 2024). The GWAS summary statistics for asthma were collected from the UK Biobank, comprising data from 56,167 asthma cases and 352,255 controls.^[23]^ Asthma cases were identified through self-reported questionnaires, hospital records coded according to the International Classification of Diseases (ICD-9 and ICD-10), and primary care records.

The Neale Lab or MRC-IEU consortium provided combined data on BMI, EA, smoking, and TDI. Summary data for pediatric asthma in the reverse MR analysis was sourced from a GWAS study by Sakae et al,^[24]^ which included 438,843 individuals and detected 24,166,696 SNPs. The information regarding endometriosis in reverse MVMR was also compiled from Ben Elsworth recently published GWAS study, which entailed 3809 cases and 45,914 controls. All individuals involved in the investigation are of European ancestry. Table 1 provides a clear overview of the datasets that comprised this study.

**Table 1:** Details of studies included in Mendelian randomization (MR) analyses.

Traits	Author	GWAS ID	Sample size (cases/controls)	Number of SNPs	Ancestry	Year	PMID
Exposure							
AAM	Loh PR	ebi-a-GCST90029036	279,470	11,971,701	European	2018	29892013
ANM	Ben Elsworth	ukb-b-17422	143,819	9851,867	European	2018	NA
AFS	Mills MC	ebi-a-GCST90000047	397,338	16,359,424	European	2021	34211149
AFB	Mills MC	ebi-a-GCST90000050	542,901	9702,772	European	2021	34211149
ALB	Ben Elsworth	ukb-b-8727	170,248	9851,867	European	2018	NA
Number of live births	Ben Elsworth	ukb-b-1209	250,782	9851,867	European	2018	NA
Number of stillbirths	Ben Elsworth	ukb-b-6412	78,879	9851,867	European	2018	NA
Number of SM	Ben Elsworth	ukb-b-419	78,700	9851,867	European	2018	NA
Hysterectomy	Ben Elsworth	ukb-b-3700	46,411/416,522	9851,867	European	2018	NA
Bilateral oophorectomy	Ben Elsworth	ukb-b-1962	19,512/443,421	9851,867	European	2018	NA
Ever used HRT	Ben Elsworth	ukb-b-18541	97,920/152,381	9851,867	European	2018	NA
Ever taken OC pill	Ben Elsworth	ukb-b-9509	205,516/44,924	9851,867	European	2018	NA
BMI	Neale	ukb-a-248	336,107	10,894,596	European	2017	NA
Age completed full time education	Neale	ukb-a-505	226,899	10,894,596	European	2017	NA
Smoking status: Current	Neale	ukb-a-225	33,928/302,096	10,894,596	European	2017	NA
TDI	Ben Elsworth	ukb-b-10011	462,464	9851,867	European	2018	NA
Pediatric asthma	Sakaue S	ebi-a-GCST90018895	27,712/ 411,131	24,166,696	European	2021	34594039
EM	Ben Elsworth	ukb-b-10903	3809/ 459,124	9851,867	European	2018	NA
Outcomes							
Asthma	Valette K	ebi-a-GCST90014325	56,167/352,255	34,551,291	European	2021	34103634

AAM = age at menarche, AFB = age at first birth, AFS = age at first sexual intercourse, ALB = age at last live birth, ANM = age at natural menopause, BMI = body mass index, EM = endometriosis, HRT = hormone replacement therapy, OC = oral contraceptive, SM = spontaneous miscarriage, TDI = Townsend deprivation index.

### 2.3. Selection and evaluation of IVs

To meet the essential assumptions of MR analysis (Fig. 1), we followed a distinct procedure for IV selection. For the majority of female reproductive traits, SNPs significantly associated with each exposure were identified at the genome-wide significance threshold (*P* < 5 × 10^−8^). For HRT and OC use – traits with fewer genome-wide significant variants – we applied a less stringent threshold (*P* < 5 × 10^−6^) to ensure adequate instrument strength, as supported by previous MR studies. SNPs linked to asthma, including pediatric asthma, were also identified at a threshold of *P* < 5 × 10^−8^ in the reverse MR analysis. To address the influence of linkage disequilibrium (LD) among the SNPs, we implemented a rigorous criterion (*r*^2^ < 0.001 and a clumping distance of 10,000 kb) to ensure the conditional independence of the selected IVs. We retained only SNPs with the most significant *P*-values.^[25]^ Additionally, we checked potential pleiotropic effects by extracting the secondary phenotype of each SNP using LDtrait Tool (https://ldlink.nih.gov/?tab=ldtrait).^[21]^ SNPs that exhibited connections with reported phenotypes were excluded from subsequent analyses. Finally, IVs were extracted from the outcome data, and data harmonization was conducted to remove SNPs with inconsistent alleles between exposure and outcome data. Palindromic SNPs with intermediate allele frequencies were also excluded to prevent strand ambiguity. The robustness of IVs was evaluated employing variance (R^2^) and *F*-statistic to mitigate the influence of weak instrument bias. The formula to calculate the *F*-statistic for each SNP is *F* = *R*^2^/(1 − *R*^2^)[(N − *K* − 1)/*K*], where N represents the sample size, *K* denotes the total number of SNPs selected for MR analysis, and *R*^2^ reflects the overall proportion of phenotypic differences explained by all the SNPs in our MR model.^[26]^ The R2 for each SNP was calculated utilizing the following formula: *R*^2^ = 2 * EAF * (1 − EAF) * β^2^, where β is the β coefficient for effect size, EAF is the effect allele frequency for each SNP.^[27]^ An *F*-statistic exceeding 10 was considered significant for the association between IVs and exposure, ensuring the results were not affected by weak instrument bias.^[28]^ Statistical power for each outcome was determined using the online tool available at https://shiny.cnsgenomics.com/mRnd/, and the full results are reported in Table 2.

**Table 2:** The results of the selected IVs strength and statistical power.

Exposures	*R*^2^ for TL (total)	Power
AAM	0.094	0.48
ANM	0.047	0.05
AFS	0.027	1.00
AFB	0.023	0.99
ALB	0.007	0.95
Number of live births	0.001	0.07
Number of stillbirths	0.0004	0.11
Number of SM	0.002	0.06
Hysterectomy	0.0002	0.07
bilateral oophorectomy	2.11346E−05	0.05
Ever used HRT	0.0009	0.40
Ever taken OC pill	0.0001	0.24

AAM = age at menarche, AFB = age at first birth, AFS = age at first sexual intercourse, ALB = age at last live birth, ANM = age at natural menopause; HRT = hormone replacement therapy, IVs = instrumental variables, OC = oral contraceptive, SM = spontaneous miscarriage.

### 2.4. Statistical analysis

In order to assess the genetic causal effects, various methodologies that included IVW, MR-Egger, weighted median, and robust adjusted profile score (RAPS) were implemented. These methods yielded reliable evidence under different circumstances, with IVW being the primary results.^[29]^ The IVW technique is an expansion of the Wald ratio estimator that utilizes meta-analytic principles. It aims to provide a unbiased estimation in an optimal scenario where all the included SNPs are assumed to be legitimate IVs without any horizontal pleiotropy or heterogeneity.^[30]^MR-Egger allows certain SNPs to impact the result by mechanisms other than exposure, which can provide a dependable and impartial estimation, even when all of the SNPs are not valid. Furthermore, the MR-Egger intercept has the capability to identify and correct for pleiotropy.^[29,31]^ Despite the fact that up to 50% of the data utilized in the study consists of invalid IVs, the weighted median method can still generate dependable estimates of the causal effects.^[32]^ The RAPS method is resistant to weak instruments and maintains robust even when systematic pleiotropy is present, and it can mitigate horizontal pleiotropy by incorporating the measurement error in the relationship between SNPs and exposure.^[33]^ Considering previous study findings^[17–20]^ as a foundation, we accounted for BMI, EA, smoking, and TDI in a MVMR analysis. This allowed us to estimated the direct impact of women’s reproductive traits independent of risk variables. The techniques we utilized to perform MVMR included IVW, MR-Lasso and MR-median.^[34]^

We further conducted a mediation analysis employing a two-step MR design to investigate whether BMI acts as a mediator in the causal pathway from AFS and AFB to asthma outcome (Fig. 1). The total effect was decomposed into 2 components: direct effects (c’ in Fig. 1), which do not involve the intermediate variables, and indirect effects mediated through the mediator, denoted as “a × b” in Figure 1.^[35]^ To determine the proportion mediated by the mediating effect, we divided the indirect effect by the total effect. Meanwhile, we computed the 95% confidence intervals using the delta method.^[36]^

In this study, a series of sensitivity analyses were carried out to confirm the stability and reproducibility of the MR results. Cochran *Q* test was employed to evaluate heterogeneity among SNPs, with a *P*-value exceeding .05 signifying no significant heterogeneity. Additionally, the MR-Egger intercept approach was utilized to estimate the degree of horizontal pleiotropy attributable to IVs. A leave-one-out analysis was also implemented to ascertain whether the MR findings were affected by any specific SNP. Furthermore, the MR-PRESSO method was applied to detect potential outlier SNPs. To adjust for multiple comparisons, the *P*-value was corrected using the Bonferroni method. The association between women’s reproductive traits and asthma was deemed statistically significant at a two-sided *P*-value below .004 (α = 0.05/12 outcomes), and suggestive when the *P*-value was under .05. MR analyses were finished using the TwoSampleMR (version 0.5.6) and MVMR (version 0.3) packages in R (version 4.3.1).

## 3. Result

### 3.1. Estimated causal effect of women’s reproductive traits on asthma

**Figure 2.**
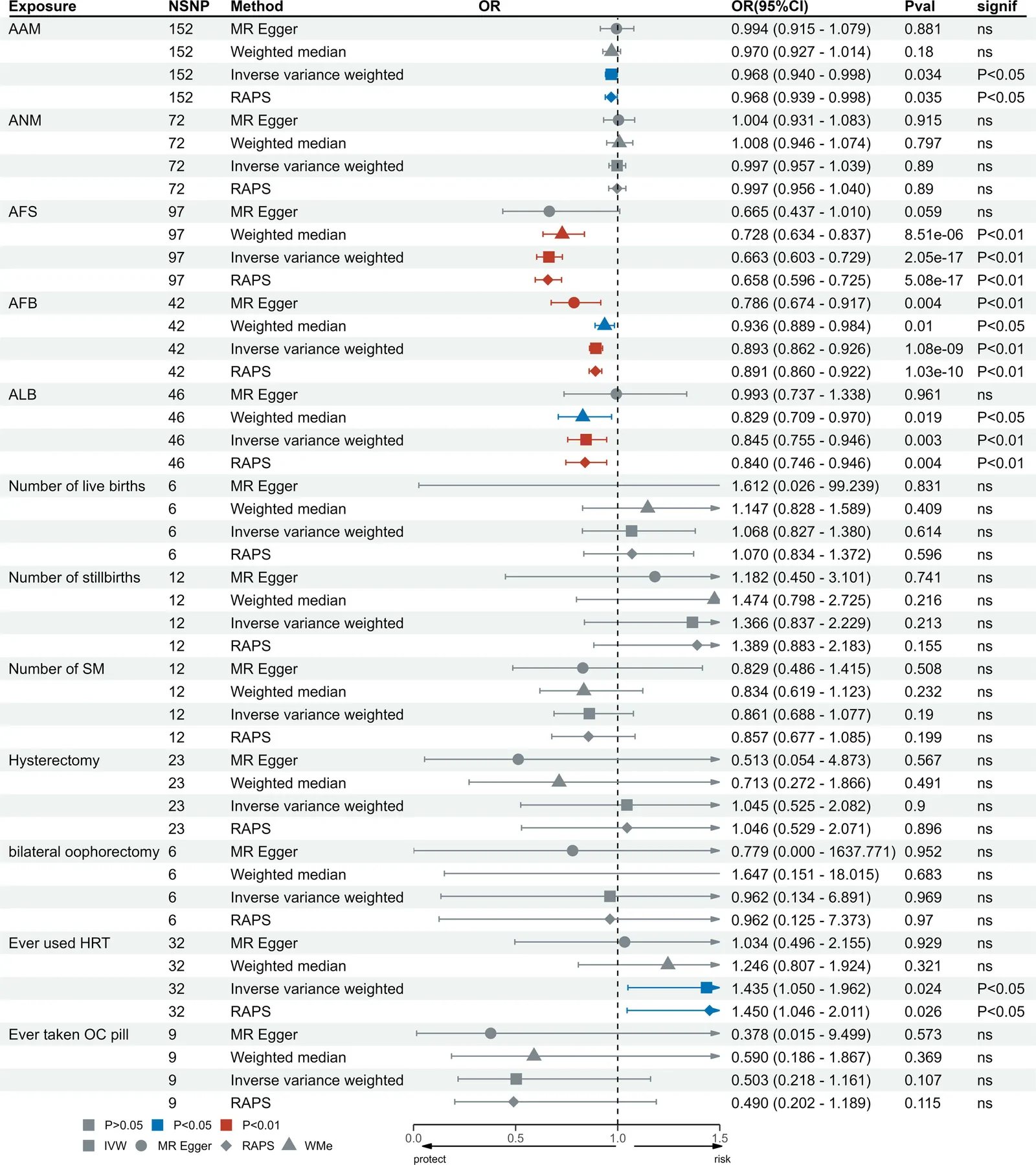
The effect of genetically determined women’s reproductive traits on asthma using UVMR. * Nominal significance (*P* < .05); ** Bonferroni-corrected significance (*P* < .004). UVMR = univariate Mendelian randomization.

We selected 152, 72, 97, 42, 46, 6, 12, 12, 23, 6, 32 and 9 SNPs as genetic instruments for AAM, ANM, AFS, AFB, ALB, number of live births, number of stillbirths, number of SM, history of hysterectomy or bilateral oophorectomy, ever taken OC pill, and ever used HRT after LD clumping and removing pleiotropic SNPs, respectively (Tables S1–S12, Supplemental Digital Content, https://links.lww.com/MD/Q156). The *F*-statistics for these genetic variants were all above the critical value of 10, suggesting a minimal risk of weak instrumental bias. Based on the application of the Bonferroni correction, our analysis revealed substantial correlations between a later AFS (OR = 0.663, 95% CI = 0.603–0.729, *P* = 2.05E−17; Bonferroni-corrected), AFB (OR = 0.893, 95% CI = 0.862–0.926, *P *= 1.08E−09; Bonferroni-corrected), and ALB (OR = 0.845, 95% CI = 0.755–0.946, *P* = 3.42E−03; Bonferroni-corrected) with a decreased risk of asthma in the IVW model. The obtained outcomes were consistent with the findings obtained from the weighted median and RAPS model. Furthermore, the IVW model showed a nominally significant negative correlation between the AAM (OR: 0.968; 95% CI: 0.94–0.998; *P* = .034; nominal significance) and asthma risk. Likewise, ever used HRT was statistically associated with an increased risk of asthma (OR: 1.435; 95% CI: 1.05–1.962; *P* = .024; nominal significance). These results aligned with those from the RAPS model. Nevertheless, the study could not identify any causal relationship between 7 additional female reproductive characteristics, namely ANM, number of live births, number of stillbirths, number of SM, hysterectomy, bilateral oophorectomy, and ever taken OC pill and asthma. Similar conclusions were drawn from the other 3 statistical models. Figure 2 demonstrates the causal link between genetic predictors of women’s reproductive traits on the risk of asthma.

**Figure 3.**
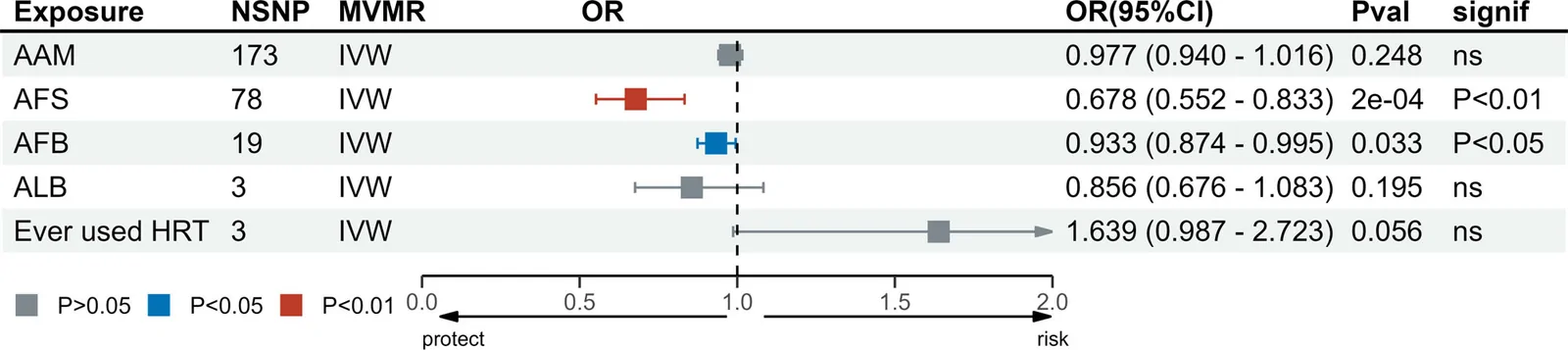
The direct effect of genetically determined women’s reproductive traits on asthma using MVMR controlled for BMI, EA, smoking, and TDI. BMI = body mass index, EA = educational attainment, MVMR = multivariate Mendelian randomization, TDI = Townsend deprivation index.

Additional genome-wide significant genetic variants related to BMI, EA, smoking, and TDI were incorporated into the MVMR analysis alongside women’s reproductive traits. After adjusting for the effects of BMI, EA, smoking, and TDI, there was also compelling evidence indicating a direct inverse association between genetically predisposed AFS (OR = 0.678, 95% CI = 0.552–0.833, *P* = 2.11E−04; Bonferroni-corrected) and AFB (OR = 0.933, 95% CI = 0.874–0.995, *P* = .033; nominal significance) and the risk of asthma (Fig. 3). In the MVMR-IVW model, the causal relationship between AAM and ever used HRT and asthma is no longer evident (Fig. 3). The MVMR-Lasso and MVMR-Egger methods provided consistent results (Table S13, Supplemental Digital Content, https://links.lww.com/MD/Q156). Notably, we found substantial proof for the direct effect of ever used HRT on asthma employing MVMR-Lasso (*P* = .002; Bonferroni-corrected) and MVMR-Egger (*P* = .02; nominal significance) methods, while no association found using MVMR-IVW method (Table S13, Supplemental Digital Content, https://links.lww.com/MD/Q156).

We conducted an analysis of BMI as a mediator in the pathway from AFS or AFB to asthma. Our findings revealed that both AFS and AFB were associated with increased BMI, which subsequently correlated with a heightened risk of asthma. As illustrated in Table 3, our study demonstrated that BMI explained 15.7% or 17.6% of the elevated risk of asthma linked to AFS (proportion mediated: 15.7%; 95% CI = 9.0%−22.4%; nominal significance) or AFB (proportion mediated: 17.6%; 95% CI = 6.0%−29.1%; nominal significance), respectively.

**Table 3:** Estimates of the effect of AFS or AFB on asthma explained by BMI.

Exposure	Mediator	Outcome	Indirect effect β (95% CI)	Proportion mediated (%) (95% CI)
AFS	BMI	Asthma	−0.065 (−0.089, −0.041)	15.7 (9.0, 22.4)
AFB	BMI	Asthma	−0.015 (−0.021, −0.009)	17.6 (6.0, 29.1)

AFB = age at first birth, AFS = age at first sexual intercourse, BMI = body mass index.

**Figure 4.**
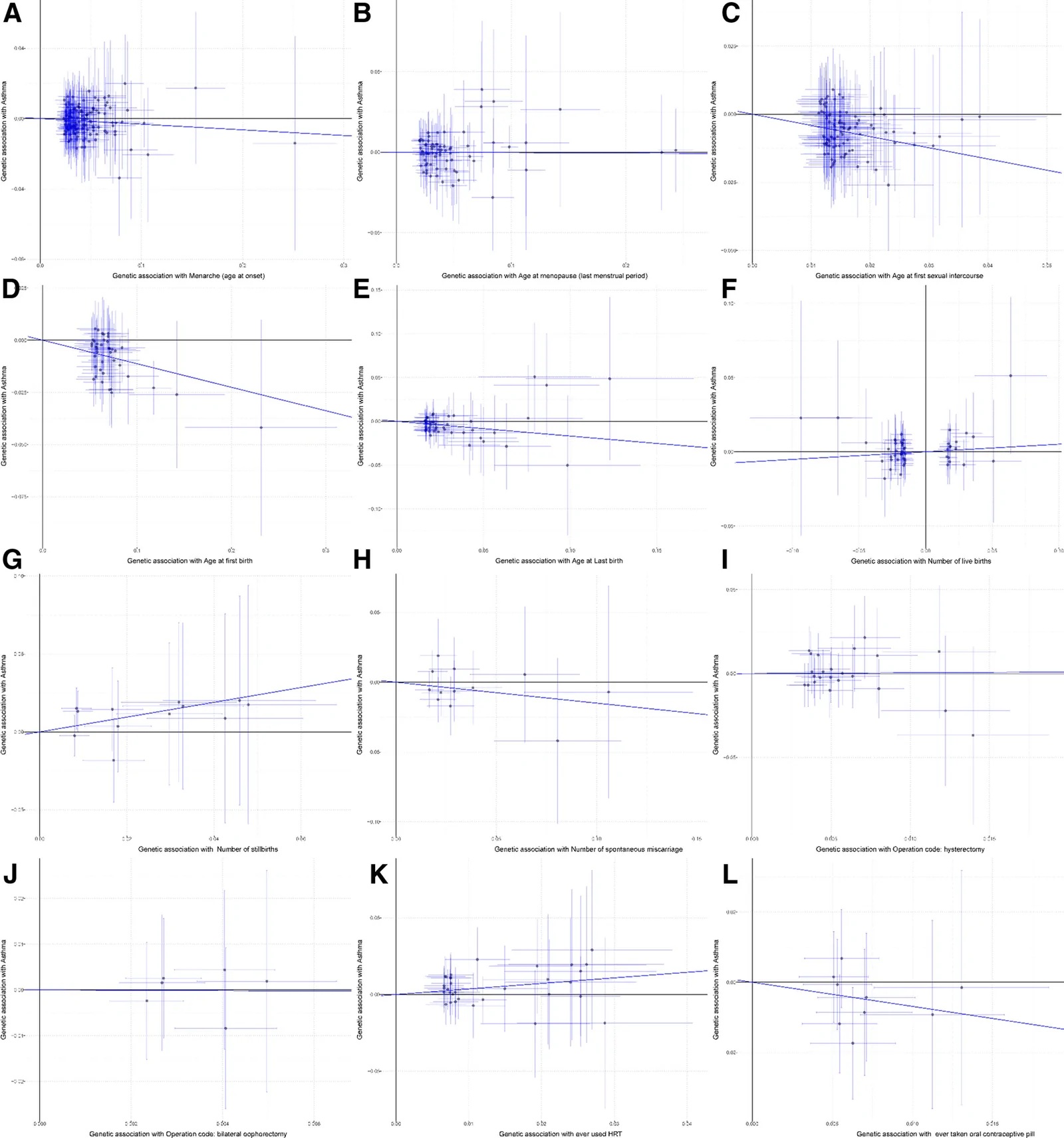
Scatter plots for MR analyses of the correlation between women’s reproductive traits on asthma in the IVW model. (A) AAM; (B) ANM; (C) AFS; (D) AFB; (E) ALB; (F) number of live births; (G) number of stillbirths; (H) number of SM; (I) hysterectomy; (J) bilateral oophorectomy; (K) ever used HRT; (L) ever taken OC pill. AAM = age at menarche, AFB = age at first birth, AFS = age at first sexual intercourse, ALB = age at last birth, ANM = age at natural menopause, MR = Mendelian randomization, HRT = hormone replacement therapy, IVW = inverse-variance weighted, SM = spontaneous miscarriage.

In the sensitivity analyses, MR-Egger regression intercepts for all exposures were close to zero with nonsignificant *P*-values (Table 4), indicating no evidence of directional pleiotropy. Similarly, Cochran *Q* statistics for both MR-Egger and inverse-variance weighted (IVW) methods showed no significant heterogeneity across SNPs. The MR-PRESSO global test did not detect any outlier SNPs for any exposure–outcome pair. Figure 4 displays scatter plots of SNP-specific associations between each female reproductive trait and asthma risk, with fitted lines representing causal estimates from MR-Egger (blue) and IVW (black) methods. For traits showing Bonferroni-corrected or nominal significance in the primary MR analysis (e.g., AFS, AFB, ALB, AAM, ever used HRT), the direction and slope of the IVW and MR-Egger lines were generally consistent, supporting the robustness of the causal estimates. No substantial leverage points or influential SNPs were apparent in the scatter plots, which is further confirmed by the leave-one-out analyses (Fig. S1 and S2, Supplemental Digital Content, https://links.lww.com/MD/Q157), where sequential removal of individual SNPs did not materially alter the overall causal estimates.

**Table 4:** Heterogeneity, horizontal pleiotropy, and MR-PRESSO tests of the associations between women’s reproductive traits and asthma.

Exposures	Pleiotropy test			Heterogeneity test						MR-PRESSO
	MR-Egger			MR-Egger			Inverse-variance weighted			Global test
	Intercept	SE	*P*	*Q*-value	*Q*-df	*Q*-pval	*Q*-value	*Q*-df	*Q*-pval	*P*-value
AAM	−0.001	0.002	.510	110.054	150	.994	110.489	151	.994	.992
ANM	−0.0004	0.002	.826	65.498	70	.630	65.546	71	.660	.685
AFS	−3.33E−05	0.003	.992	87.468	95	.696	87.469	96	.721	.797
AFB	0.009	0.005	.102	43.897	40	.310	46.980	41	.241	.262
ALB	−0.004	0.003	.259	33.642	44	.871	34.951	45	.860	.87
Number of live births	−0.011	0.055	.854	5.517	4	.238	5.570	5	.350	.363
Number of stillbirths	0.006	0.006	.322	8.653	10	.565	9.738	11	.554	.601
Number of SM	0.001	0.007	.882	10.581	10	.391	10.606	11	.477	.531
Hysterectomy	0.004	0.006	.522	22.958	21	.346	23.423	22	.378	.372
Bilateral oophorectomy	0.0007	0.013	.958	1.455	4	.834	1.459	5	.918	.915
Ever used HRT	0.004	0.004	.341	23.717	30	.785	24.652	31	.783	.785
Ever taken OC pill	0.002	0.011	.862	7.231	7	.405	7.265	8	.508	.557

AAM = age at menarche, AFB = age at first birth, AFS = age at first sexual intercourse, ALB = age at last live birth, ANM = age at natural menopause, HRT = hormone replacement therapy, MR = Mendelian randomization, OC = oral contraceptive, SM = spontaneous miscarriage.

### 3.2. Estimated causal effect of asthma on women’s reproductive traits

We identified 42, 45, 46, 47, 49, 50, 48, 51, 51,52, 53 and 50 SNPs as IVs for asthma, to evaluate its associations with AAM, ANM, AFS, AFB, ALB, number of live births, number of stillbirths, number of SM, history of hysterectomy or bilateral oophorectomy, ever taken OC pill, and ever used HRT after LD clumping and removing pleiotropic SNPs, respectively, as detailed in Tables S14 to S25 (Supplemental Digital Content, https://links.lww.com/MD/Q156). To elucidate the association between asthma and female reproductive traits, given that most female reproductive behaviors manifest in adulthood, additional MR analysis was performed, focusing on childhood asthma and various female reproductive characteristics. Information regarding the pertinent IVs is provided in Tables S26 and S37 (Supplemental Digital Content, https://links.lww.com/MD/Q156). All genetic variations taken into the reverse analysis possessed *F*-statistics that exceeded the essential threshold of 10, indicating a low risk of weak instrumental bias.

**Figure 5.**
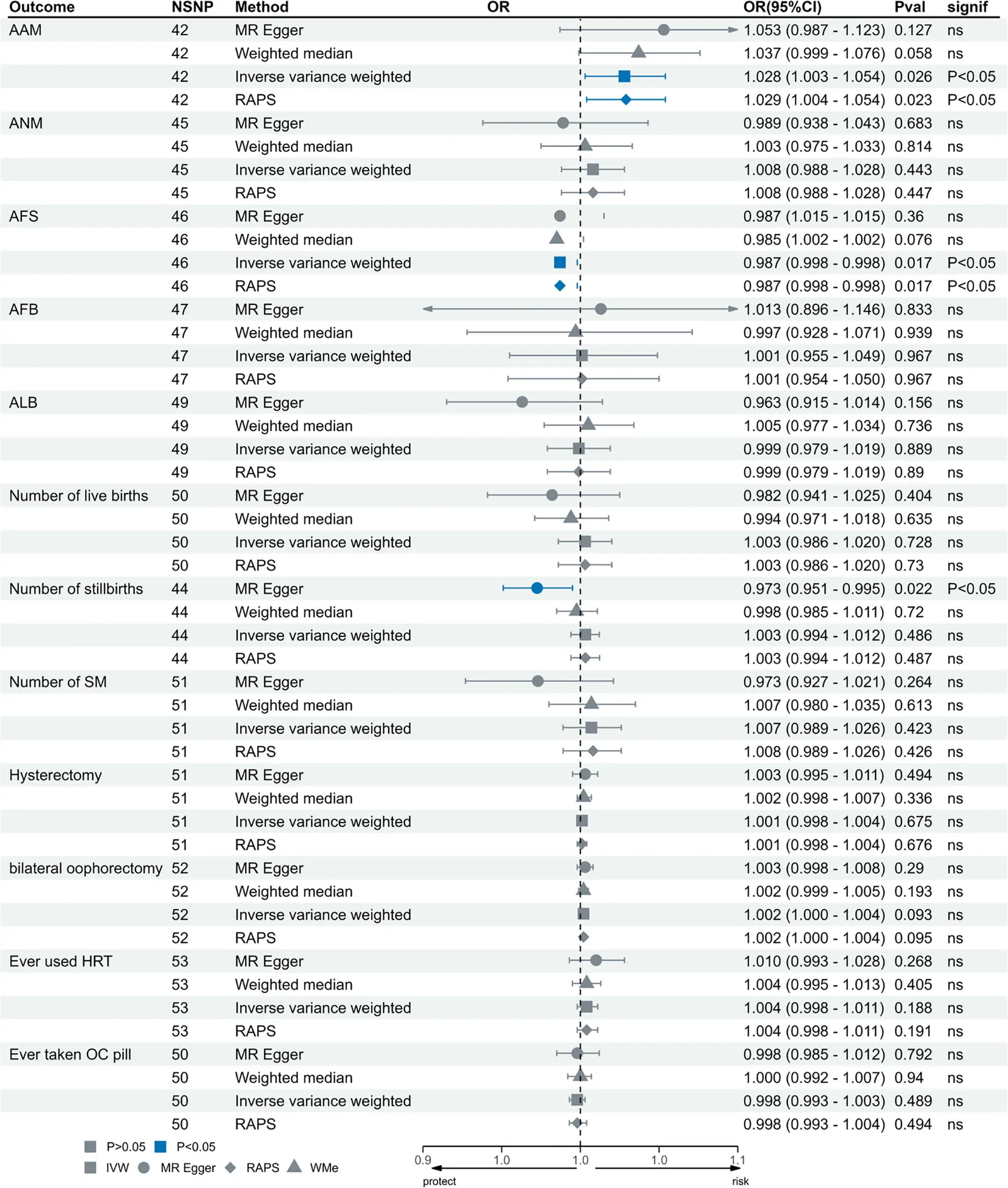
The effect of genetically determined asthma on women’s reproductive traits using UVMR. UVMR = univariate Mendelian randomization.

Asthma considerably postponed AAM, according to the IVW model (OR: 1.028; 95% CI: 1.003–1.054; *P* = .026; nominal significance). Similarly, the RAPS model yielded comparable findings. However, this association ceased to be significant after MVMR adjustments for BMI and EA (OR: 0.989; 95% CI: 0.939–1.041; *P* = .673). In a similar vein, no conclusive evidence connected childhood asthma to AAM (OR: 1.018; 95% CI: 0.99–1.047; *P* = .202). The IVW model suggested a nominal negative correlation between asthma (OR: 0.987; 95% CI: 0.976–0.997; *P* = .017; nominal significance) or pediatric asthma (OR: 0.986; 95% CI: 0.975–0.998; *P* = .018; nominal significance) and AFS. The RAPS model produced similar results. Yet, these relationships were nullified after adjusting for BMI and EA (OR: 0.989; 95% CI: 0.969–1.01; *P* = .313) (OR: 0.99; 95% CI: 0.969–1.012; *P* = .375). Although asthma in children was initially linked to an increased risk of hysterectomy (OR: 1.005; 95% CI: 1.002–1.008; *P* = 9.68E−04; Bonferroni-corrected) and oophorectomy (OR: 1.002; 95% CI: 1–1.004; *P* = .048; nominal significance), these associations disappeared when endometriosis is taken into account using MVMR adjustment (OR: 1.004; 95% CI: 0.998–1.009; *P* = .185) (OR: 1.002; 95% CI: 0.999–1.004; *P* = .23). Across all 4 statistical models employed, no causal relationship was established between genetic predisposition to asthma or childhood asthma and other female reproductive behaviors. Figure 5 depicts the causal connections between genetic predictors of asthma and women’s reproductive traits. The effect of genetically determined pediatric asthma on women’s reproductive traits was shown in Figure S3 (Supplemental Digital Content, https://links.lww.com/MD/Q157). Figure S4 (Supplemental Digital Content, https://links.lww.com/MD/Q157) displays the outcomes of the MVMR analysis. Cochran *Q* test revealed no evidence of heterogeneity, and the MR-Egger intercept test only detected signs of horizontal pleiotropy in the MR analysis between asthma and the number of stillbirths as well as childhood asthma and AAM. Furthermore, the MR-PRESSO analyses identified no outliers among the SNPs. Comprehensive findings from the sensitivity analyses are detailed in Tables S38 and S39 (Supplemental Digital Content, https://links.lww.com/MD/Q156). Figures S5 to S8 (Supplemental Digital Content, https://links.lww.com/MD/Q157) present scatter plots detailing the associations between asthma, including pediatric asthma, and women’s reproductive traits. The leave-one-out plots indicated that the specific SNPs were not expected to have an impact on causal estimates (Figs. S9–S12, Supplemental Digital Content, https://links.lww.com/MD/Q157).

## 4. Discussion

In our study, we employed MR analysis to explore the bidirectional causal relationship between 12 female reproductive characteristics and asthma. For clarity, all statistical significances reported below follow the definitions in the Results section: Bonferroni-corrected (*P* < .004) and nominal significance (0.004 ≤ *P* < .05). Our findings revealed a significant association between the genetic predictors of key reproductive events and the risk of asthma.

Following Bonferroni correction, our UVMR results indicated that earlier genetically predicted AFS and AFB were associated with an increased risk of asthma, and this remained significant in the MVMR model after adjusting for BMI, EA, smoking, and TDI. Mediation analysis suggested that BMI partially mediates these associations. These findings imply that delaying AFS may exert a protective effect on asthma risk. One possible explanation is that premature sexual activity may lead to earlier and more frequent activation of sex hormones such as estrogen, which can influence airway structure, inflammatory responses, and smooth muscle function, thereby contributing to asthma onset.^[37]^ In addition, early AFS is often accompanied by unprotected sexual behavior, hormonal contraceptive use, and increased psychosocial stress, which have been linked to asthma in observational studies.^[38–40]^ For example, a cross-sectional study reported a higher risk of asthma attacks among women with a history of forced sexual intercourse (OR = 3.67, 95% CI, 1.76–7.69).^[41,42]^ While these mechanisms are biologically plausible, the current evidence remains largely indirect and derived from observational data; thus, further longitudinal and mechanistic studies are warranted to substantiate these hypotheses.^[43]^

Correspondingly, an early AFS might lead to a younger AFB. A clinical study from South Korea demonstrated that women under 20 years old at the time of their first childbirth had a significantly higher association with asthma diagnosis compared to women aged 25 to 29 (OR = 1.81, 95% CI: 1.14–2.89).^[44]^ A prospective cohort study conducted in Europe also indicated that an early AFB (≤ 20 years old) corresponds to an increased risk of asthma.^[10]^ In line with prior research, our study found an interaction between an earlier AFB and a higher chance of developing asthma. While the precise mechanisms underlying this relationship are not fully understood, it may be explained from 3 aspects. Firstly, changes in the maternal immune system brought on by normal pregnancy are linked to the primary immune inflammatory response seen in asthma, with allergic airway inflammation mediated by Th2 phenotype lymphocytes involving cytokines such as IL-4 and IL-5.^[45]^ During pregnancy, to maintain maternal–fetal immune tolerance, there is a shift toward Th2 dominance, resulting in a relatively immunosuppressive state,^[46]^ which may heighten susceptibility to allergic reactions. Secondly, elevated estrogen levels during pregnancy can act on multiple airway cell types to promote mucus hypersecretion, airway remodeling, and hyperresponsiveness, potentially contributing to asthma onset.^[47]^ Finally, environmental or socioeconomic factors may also play a role; for example, younger mothers may experience greater exposure to indoor air pollutants from cooking fumes or coal burning, both of which have been associated with increased asthma risk.^[48]^ Notably, the protective effects of delayed AFS and AFB on asthma risk were attenuated after adjustment for BMI, with mediation analysis indicating that BMI accounted for approximately 15.7% and 17.6% of these associations, respectively. This underscores that active weight management and BMI reduction may confer broad benefits in asthma prevention.

The findings from our UVMR investigation revealed a significant association between an earlier ALB and the occurrence of asthma (adjusted for Bonferroni correction). Additionally, there was a nominal association between an earlier AAM, prior use of HRT, and asthma. However, in the MVMR model, the correlations between ALB, AAM, HRT, and asthma did not persist after correcting for confounding variables.

Currently, there is limited research exploring the connection between ALB and asthma. Previous MR study has demonstrated a positive correlation between ALB and the total number of live births.^[49]^ An earlier ALB suggests a reduction in the overall years of fertility and a decrease in the number of live births. Therefore, it is pertinent to discuss these 2 reproductive behaviors in conjunction with each other. The research exploring the relationship between ALB or the number of live births and asthma has led to conflicting conclusions. For instance, the results of a cohort study conducted in 2014 indicated a significant decrease in the adjusted risk of death from asthma with increasing parity.^[9]^ This finding suggests that having more live births may exert a protective effect against severe asthma. Conversely, a prospective cohort study found that an increased incidence of asthma has been traced to a shorter reproductive age (≤ 32 years old). Furthermore, the results of a 2016 clinical study revealed a significantly higher incidence of asthma in women who had given birth to more than 5 children (OR = 1.81, 95% CI: 1.14–2.89). Nevertheless, the association lost significance when confounding variables such income, education, and BMI were taken into account.^[44]^ Our research findings align with this, our UVMR results did not provide genetic evidence supporting a causal link between the number of live births and asthma. When BMI, smoking, education, and TDI were considered in the MVMR model, the strong connection between early AFB and asthma incidence that was evident in the UVMR results vanished. These outcomes suggest that the relationship between ALB or number of live births and asthma is contentious and warrants further investigation. Socioeconomic factors, such as education and income, which are in relation to ALB or number of live births, have been identified as risk factors for asthma.^[50]^ This might explain the association seen in observational studies between ALB or live births and asthma when socioeconomic factors are not taken into consideration.

The linkage between AAM and asthma has been widely studied but remains controversial. Some cross-sectional and cohort studies have reported that early menarche increases the risk of asthma in childhood or adulthood,^[51,52]^ while a recent prospective cohort suggested a U-shaped relationship, with both early (≤ 11 years) and late (≥ 15 years) menarche associated with elevated adult asthma risk.^[10]^ Conversely, MR studies have suggested a protective effect of later menarche on asthma risk, although these analyses did not incorporate MVMR.^[53,54]^ Observational studies have also shown that early menarche is associated with higher BMI, which in turn is strongly related to asthma risk (OR = 1.50, 95% CI: 1.18–1.90).^[6]^ Our findings align with this, as the nominal association between early AAM and asthma in UVMR was no longer significant after adjusting for BMI in MVMR.

HRT use is common in the management of menopausal symptoms, yet its association with asthma risk remains debated. Evidence from multiple studies indicates that certain HRT subtypes and prolonged use may increase asthma risk, while discontinuation of HRT has been associated with cessation of asthma treatment.^[55–58]^ Some research has found that estrogen-only therapy is linked to a higher likelihood of asthma attacks,^[59]^ and both estrogen-only and combined estrogen–progesterone therapy have been associated with increased asthma risk, whereas progesterone-only therapy may have a protective effect.^[60]^ In our UVMR analysis, HRT showed a nominal association with increased asthma risk; however, results were inconsistent across MVMR methods. Specifically, significance was retained in the Lasso and Egger models after adjustment for BMI and smoking, but not in the MVMR-IVW model. Such discrepancies may be attributable to methodological differences – IVW assumes all genetic instruments are valid, whereas Lasso and Egger are more robust to horizontal pleiotropy – and to the relatively small number of SNPs and low total *R*^2^ for the HRT instrument set, which can reduce statistical power. Additionally, the absence of GWAS data stratified by HRT regimen or duration limits the ability to perform subtype-specific MR analyses. These findings should therefore be interpreted with caution. Until more granular data are available, clinicians should consider asthma risk when initiating HRT, particularly in patients with respiratory comorbidities, and monitor symptoms closely.^[61,62]^

Menopause has a complex relationship with asthma. Observational studies have reported a U-shaped association between ANM and adult asthma risk, with both early (≤ 46 years) and late (≥ 55 years) menopause linked to increased risk,^[10]^ and have suggested that surgical menopause may further elevate susceptibility.^[63]^ However, our MR analysis found no causal association between ANM or surgical menopause (including hysterectomy and bilateral oophorectomy) and asthma. The discrepancy from observational findings may reflect residual confounding, such as obesity or shared genetic predisposition. For example, genetic overlap between endometriosis and asthma could partly explain the reported link between surgical menopause and asthma in unadjusted studies.^[64]^ Likewise, we found no genetic evidence supporting a causal relationship between stillbirths or miscarriages and asthma, although previous studies have suggested associations for higher numbers of such events (≥ 2). These discrepancies may also arise from confounding by comorbidities or other risk factors. Given that our analyses were based on a limited number of SNPs for these traits, further MR studies using larger datasets are warranted.

Clinical studies have reported inconsistent associations between asthma and AAM: some found delayed menarche in asthmatic patients, possibly due to inflammation affecting reproductive function,^[65]^ whereas others observed earlier menarche in women with childhood asthma.^[52]^ In our reverse MR analysis, asthma appeared to delay AAM, but this association was not significant after adjusting for BMI and EA in MVMR, and no causal link was found for childhood asthma. Similarly, nominal inverse associations between asthma or childhood asthma and age at first sexual intercourse (AFS) disappeared after BMI and EA adjustment. Childhood asthma was also associated with a higher risk of hysterectomy or oophorectomy in IVW, but this attenuated after MVMR adjustment for endometriosis, suggesting that prior observational findings may reflect residual confounding.

To the best of our understanding, this study represents the inaugural application of the MR framework to assess the genetic causal relationship between female reproductive characteristics and asthma. The MR approach offers several merits. Primarily, this methodology successfully mitigates the influence of possible genetic variants that are frequently linked to confounding factors in epidemiological investigations. Additionally, the substantial sample size bolsters the statistical strength of our analysis, underpinning the established correlations. All selected IVs had *F*-statistics exceeding the conventional threshold of 10, indicating sufficient instrument strength. For key exposures such as AFS, AFB, and ALB, the statistical power exceeded 0.95, supporting the robustness and reliability of the causal estimates. Furthermore, we performed an exhaustive sensitivity analysis to affirm the reliability of our findings. MVMR was employed to adjust for factors such as BMI, smoking, EA, and TDI, thereby elucidating the direct impact of female reproductive characteristics on asthma. Nonetheless, the study is not without limitations. Reliance on aggregated data from the GWAS database precludes evaluation of the nonlinear relationships between female reproductive traits and asthma. The inability to access age-stratified or medication regimen-stratified data constrains deeper investigation into the more nuanced associations between HRT and OC, and asthma, including variances in estrogen’s mechanism of action on asthma at different life stages. Although the *F*-statistics for all exposures exceeded 10, several traits – such as ANM, HRT, and OC use – had relatively low statistical power (<0.40), primarily due to limited *R*^2^ and a small number of associated SNPs. Such limitations may increase the risk of false-positive findings, and the corresponding results are therefore interpreted with caution and are not emphasized in our primary conclusions. Finally, the predominance of participants of European ancestry reduces population heterogeneity and minimizes stratification bias; however, it may limit the generalizability of our findings to other ethnic groups, and future studies incorporating multi-ethnic GWAS data are warranted.

## 5. Conclusion

Our MR analyses suggest that earlier AFS and AFB are associated with increased asthma risk, consistent with a potential causal effect indicated by genetic evidence. BMI may partially mediate these relationships, implying that modifiable factors related to reproductive timing could influence asthma susceptibility. These findings warrant further investigation to validate the causal pathways and to clarify the underlying biological mechanisms.

## Acknowledgments

We would like to thank the researchers and study participants for their contributions.

## Author contributions

**Conceptualization:** Dan Sun.

**Investigation:** Dan Sun.

**Supervision:** Xinyu Han.

**Visualization:** Xingjia Wang.

**Writing – review & editing:** Xingjia Wang.

## Supplementary Material
