# Supplementary material for: Exploring the causal relationship between female reproductive traits and asthma: A bidirectional two-sample Mendelian randomization study in a European population
Source: Medicine (Baltimore). 2025 Sep 26;104(39):e44720. doi: 10.1097/MD.0000000000044720 (PMC13593263; doi:10.1097/MD.0000000000044720)

Supplementary Figure 1. The "leave-one-out" plots of the causality between female reproductive traits and asthma. (A) AAM; (B) ANM; (C) AFS; (D) AFB; (E) ALB; (F) Number of live births.

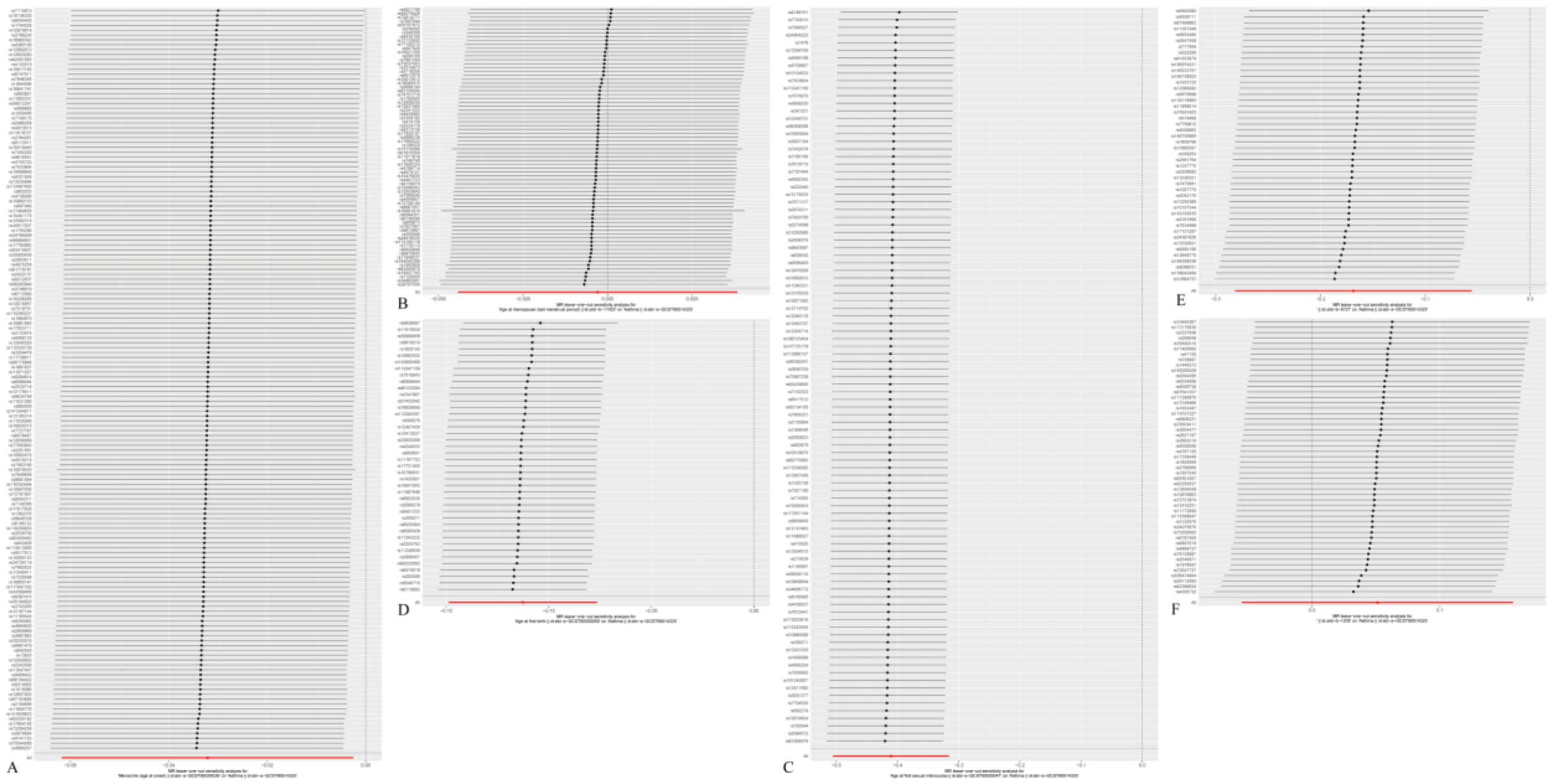

Supplementary Figure 2. The "leave-one-out" plots of the causality between female reproductive traits and asthma. (A) Number of stillbirths; (B) Number of SM; (C) Hysterectomy; (D) Bilateral oophorectomy; (E) Ever used HRT; (F) Ever taken OC pill.

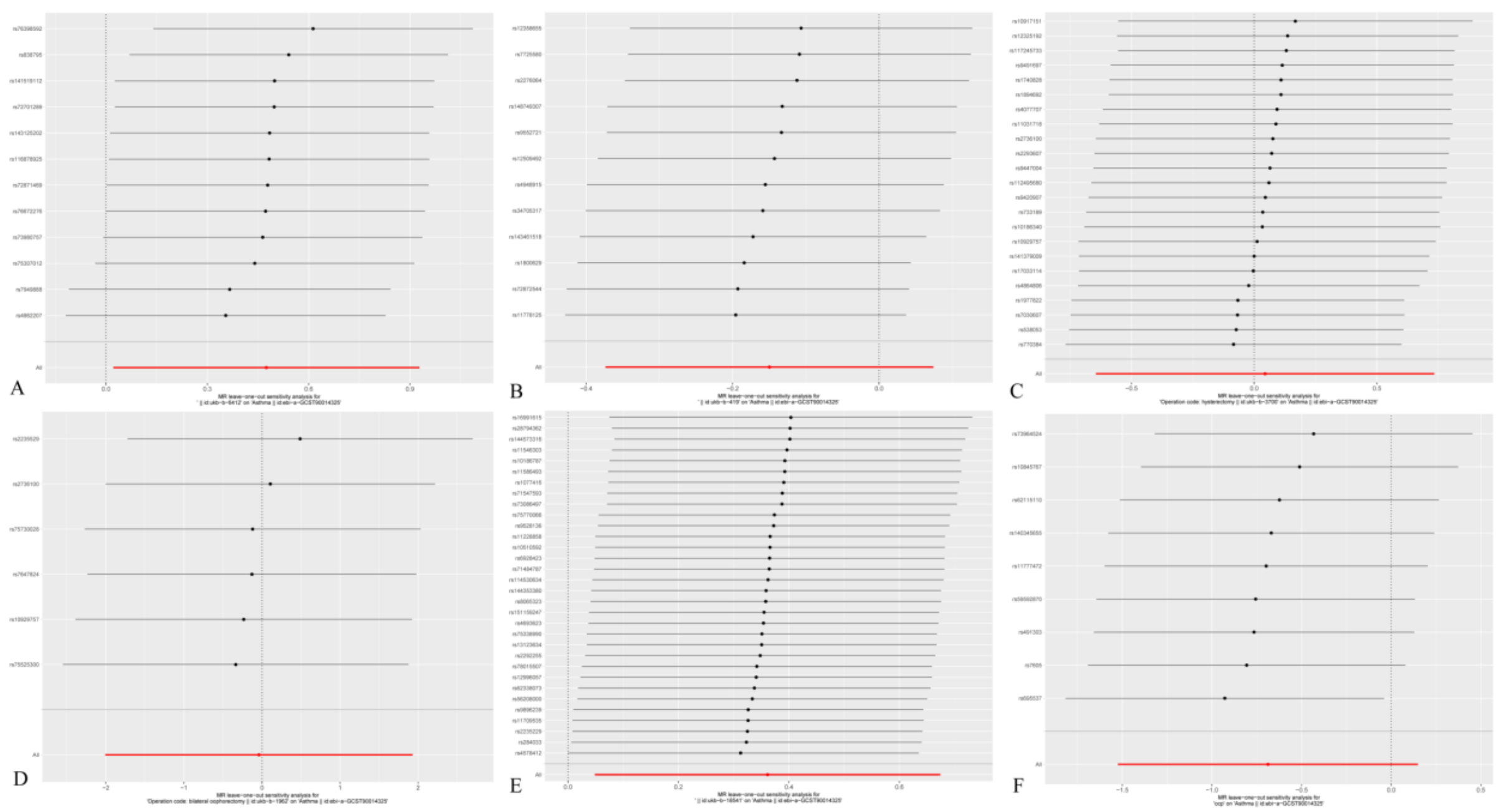

Supplementary Figure 3. The effect of genetically determined pediatric asthma on women's reproductive traits using UVMR.

Outcome	NSNP	Method	OR(95%CI)	Pval	signif
AAM	21	MR Egger	0.932 (0.857 - 1.014)	0.119	ns
	21	Weighted median	1.002 (0.965 - 1.040)	0.913	ns
	21	Inverse variance weighted	1.018 (0.990 - 1.047)	0.202	ns
	21	RAPS	1.019 (0.993 - 1.045)	0.163	ns
ANM	22	MR Egger	0.936 (0.878 - 0.997)	0.055	ns
	22	Weighted median	0.968 (0.939 - 0.997)	0.032	P<0.05
	22	Inverse variance weighted	0.994 (0.973 - 1.014)	0.545	ns
	22	RAPS	0.994 (0.973 - 1.015)	0.543	ns
AFS	21	MR Egger	1.005 (0.970 - 1.041)	0.8	ns
	21	Weighted median	0.990 (0.974 - 1.006)	0.214	ns
	21	Inverse variance weighted	0.986 (0.975 - 0.998)	0.018	P<0.05
	21	RAPS	0.986 (0.975 - 0.998)	0.019	P<0.05
AFB	26	MR Egger	0.911 (0.779 - 1.064)	0.251	ns
	26	Weighted median	0.984 (0.918 - 1.055)	0.653	ns
	26	Inverse variance weighted	0.975 (0.930 - 1.023)	0.303	ns
	26	RAPS	0.975 (0.929 - 1.023)	0.306	ns
ALB	25	MR Egger	0.946 (0.894 - 1.000)	0.061	ns
	25	Weighted median	0.981 (0.957 - 1.006)	0.138	ns
	25	Inverse variance weighted	0.983 (0.966 - 1.000)	0.051	ns
	25	RAPS	0.983 (0.966 - 1.000)	0.052	ns
Number of live births	25	MR Egger	0.983 (0.932 - 1.038)	0.548	ns
	25	Weighted median	1.007 (0.982 - 1.033)	0.587	ns
	25	Inverse variance weighted	1.005 (0.988 - 1.022)	0.595	ns
	25	RAPS	1.005 (0.987 - 1.022)	0.597	ns
Number of stillbirths	26	MR Egger	0.997 (0.966 - 1.028)	0.833	ns
	26	Weighted median	0.990 (0.978 - 1.003)	0.142	ns
	26	Inverse variance weighted	0.993 (0.984 - 1.002)	0.126	ns
	26	RAPS	0.993 (0.984 - 1.002)	0.105	ns
Number of SM	27	MR Egger	0.957 (0.890 - 1.030)	0.253	ns
	27	Weighted median	1.003 (0.976 - 1.030)	0.851	ns
	27	Inverse variance weighted	1.000 (0.981 - 1.020)	0.974	ns
	27	RAPS	1.000 (0.981 - 1.020)	0.974	ns
Hysterectomy	25	MR Egger	1.003 (0.994 - 1.013)	0.503	ns
	25	Weighted median	1.006 (1.001 - 1.010)	0.015	P<0.05
	25	Inverse variance weighted	1.005 (1.002 - 1.008)	0.001	P<0.01
	25	RAPS	1.005 (1.002 - 1.009)	0.001	P<0.01
bilateral oophorectomy	28	MR Egger	1.000 (0.993 - 1.006)	0.925	ns
	28	Weighted median	1.002 (0.999 - 1.006)	0.104	ns
	28	Inverse variance weighted	1.002 (1.000 - 1.004)	0.049	P<0.05
	28	RAPS	1.002 (1.000 - 1.004)	0.051	ns
Ever used HRT	29	MR Egger	1.000 (0.979 - 1.022)	0.976	ns
	29	Weighted median	1.006 (0.997 - 1.016)	0.2	ns
	29	Inverse variance weighted	1.004 (0.997 - 1.010)	0.289	ns
	29	RAPS	1.004 (0.997 - 1.011)	0.292	ns
Ever taken OC pill	26	MR Egger	1.001 (0.979 - 1.023)	0.949	ns
	26	Weighted median	0.995 (0.987 - 1.004)	0.273	ns
	26	Inverse variance weighted	0.996 (0.991 - 1.002)	0.245	ns
	26	RAPS	0.996 (0.990 - 1.002)	0.247	ns

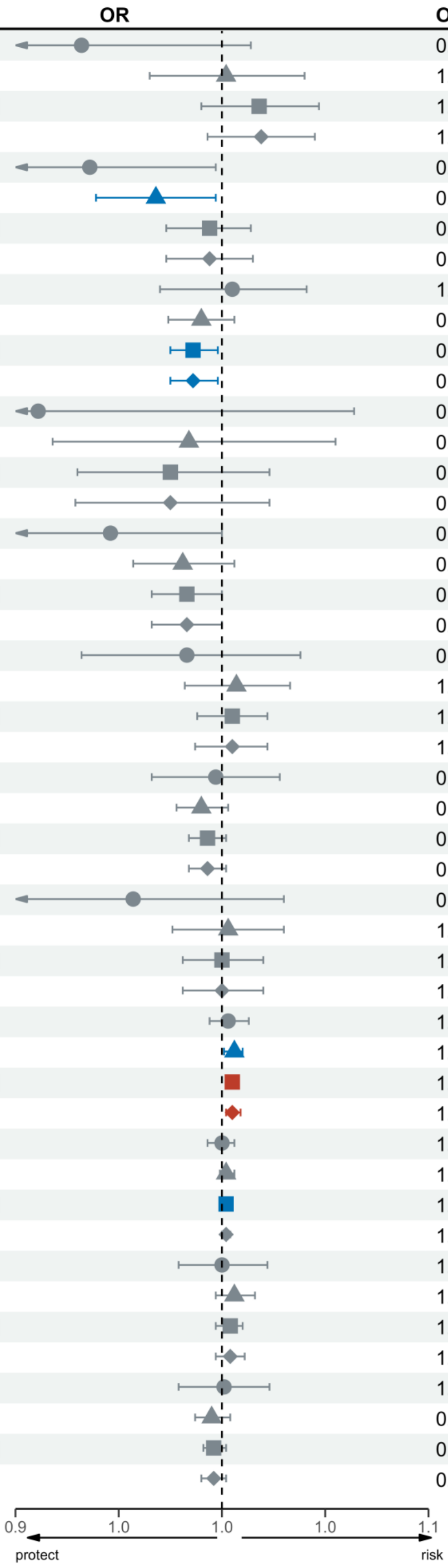

Supplementary Figure 4. The direct effect of genetically determined pediatric asthma on women's reproductive traits using MVMR controlled for BMI, EA, and EM.

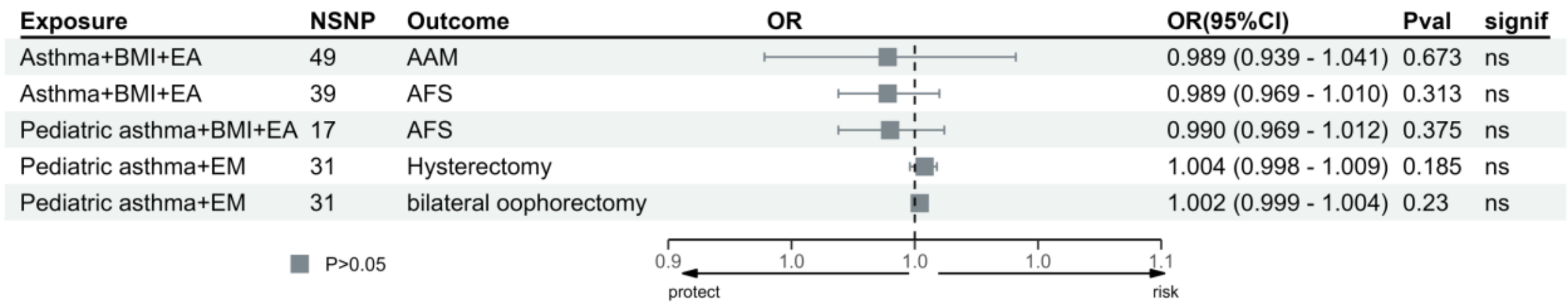

Supplementary Figure 5. Scatter plots for MR analyses of the correlation between asthma on women's reproductive traits in the IVW model. (A) AAM; (B) ANM; (C) AFS; (D) AFB; (E) ALB; (F) Number of live births.

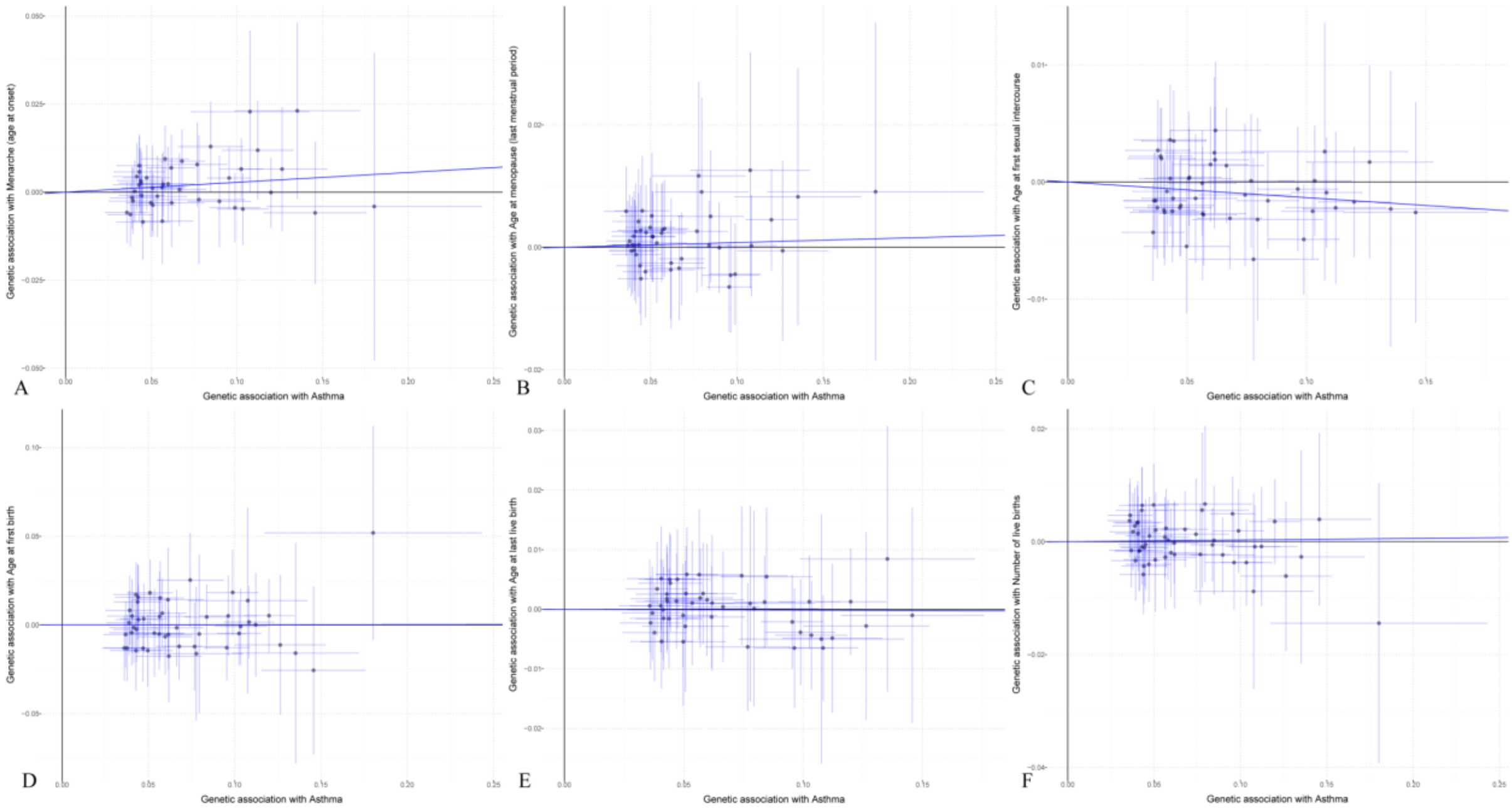

Supplementary Figure 6. Scatter plots for MR analyses of the correlation between asthma on women's reproductive traits in the IVW model. (A) Number of stillbirths; (B) Number of SM; (C) Hysterectomy; (D) Bilateral oophorectomy; (E) Ever used HRT; (F) Ever taken OC pill.

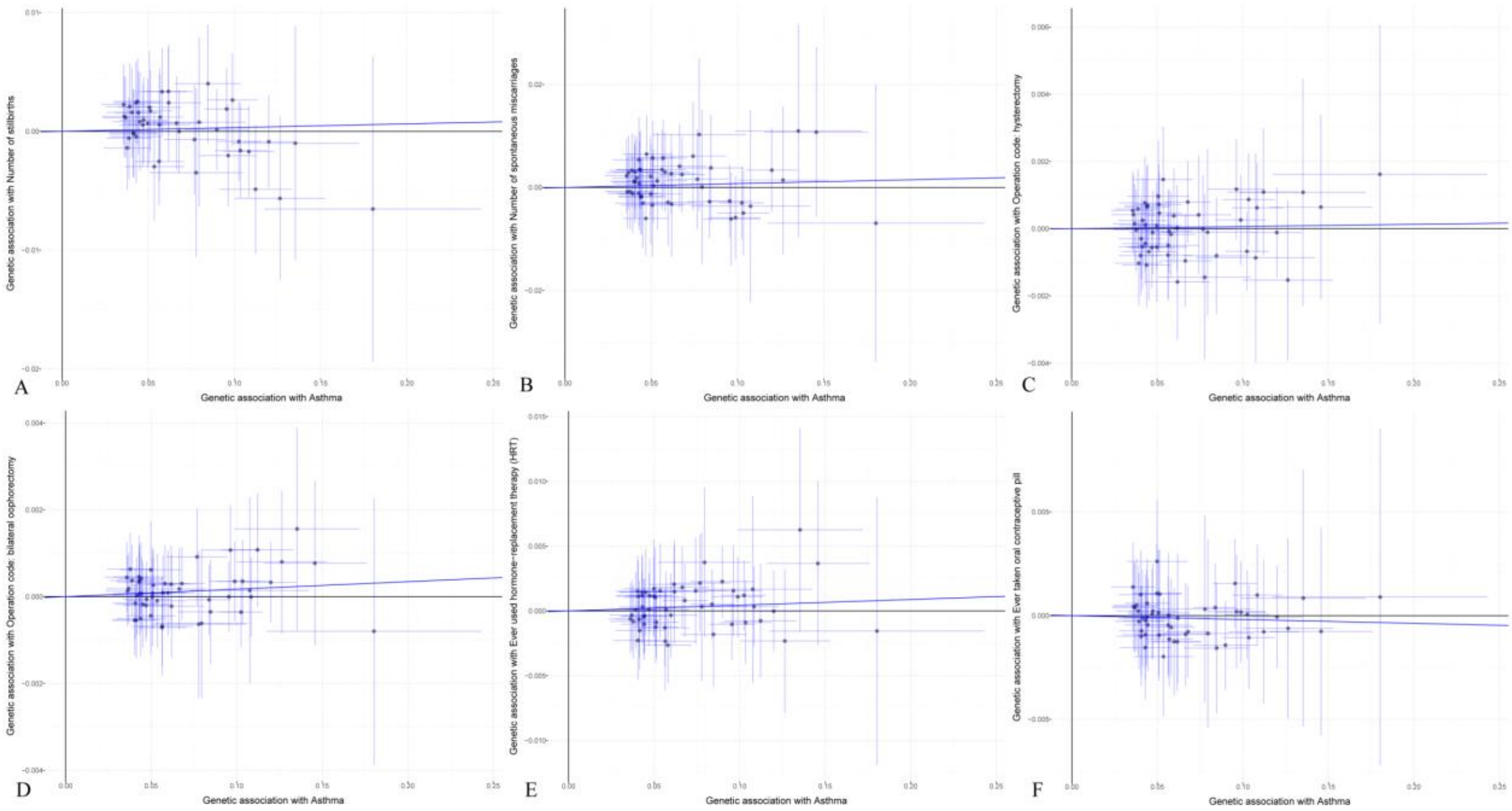

Supplementary Figure 7. Scatter plots for MR analyses of the correlation between pediatric asthma on women's reproductive traits in the IVW model. (A) AAM; (B) ANM; (C) AFS; (D) AFB; (E) ALB; (F) Number of live births.

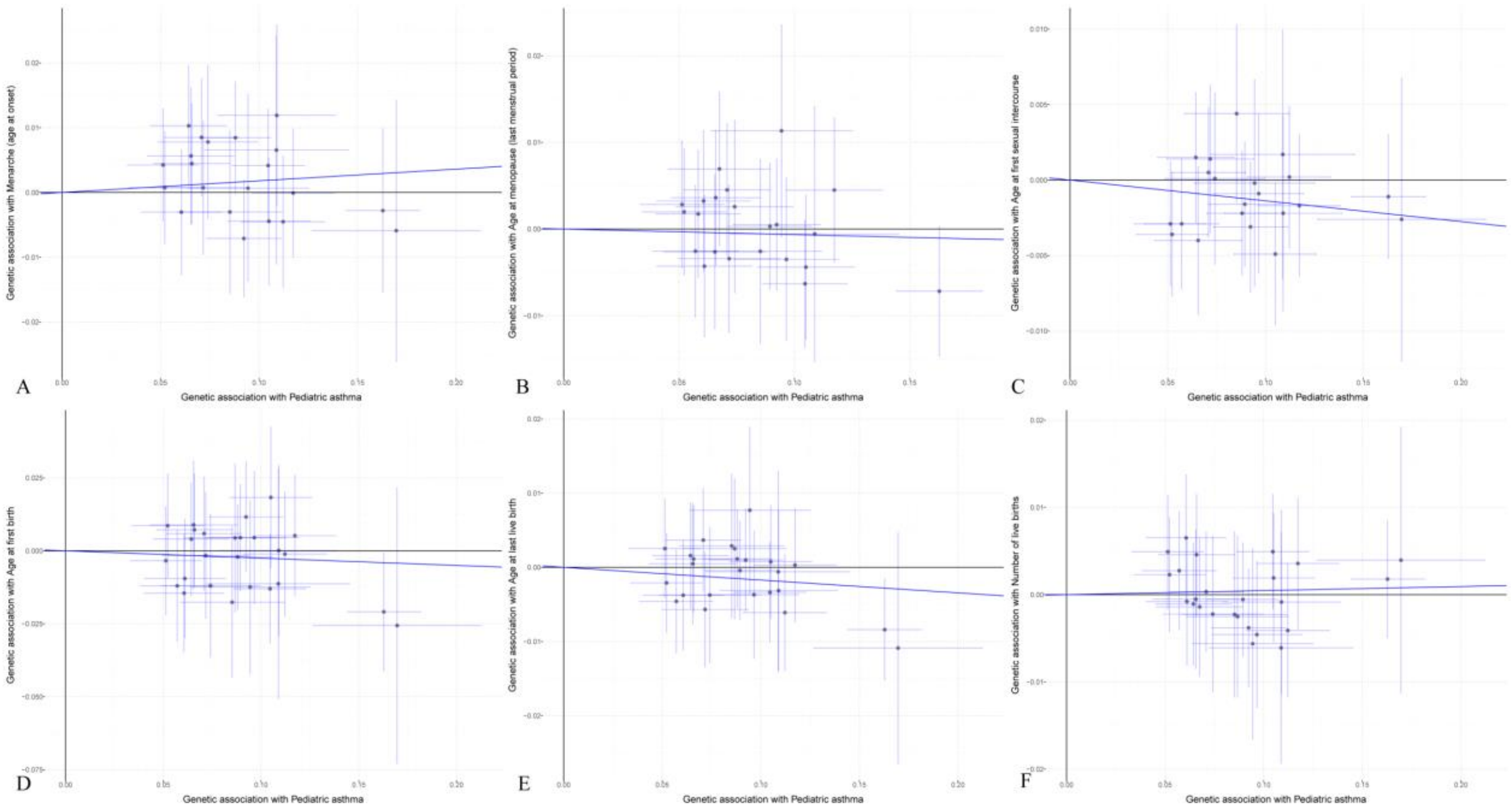

Supplementary Figure 8. Scatter plots for MR analyses of the correlation between pediatric asthma on women's reproductive traits in the IVW model. (A) Number of stillbirths; (B) Number of SM; (C) Hysterectomy; (D) Bilateral oophorectomy; (E) Ever used HRT; (F) Ever taken OC pill.

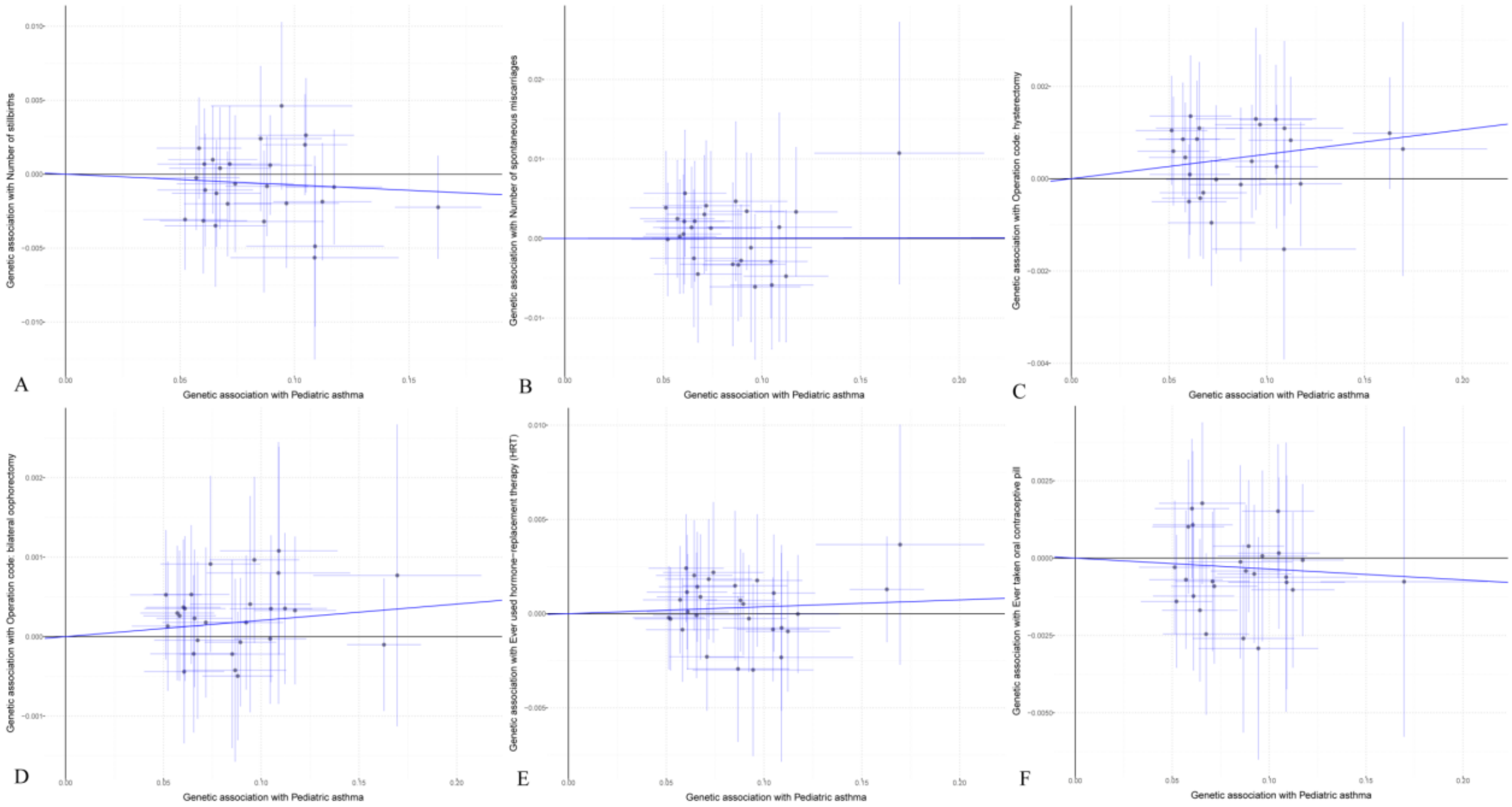

Supplementary Figure 9. The “leave-one-out” plots of the causality between asthma on women's reproductive traits**.** (A) AAM; (B) ANM; (C) AFS; (D) AFB; (E) ALB; (F) Number of live births.

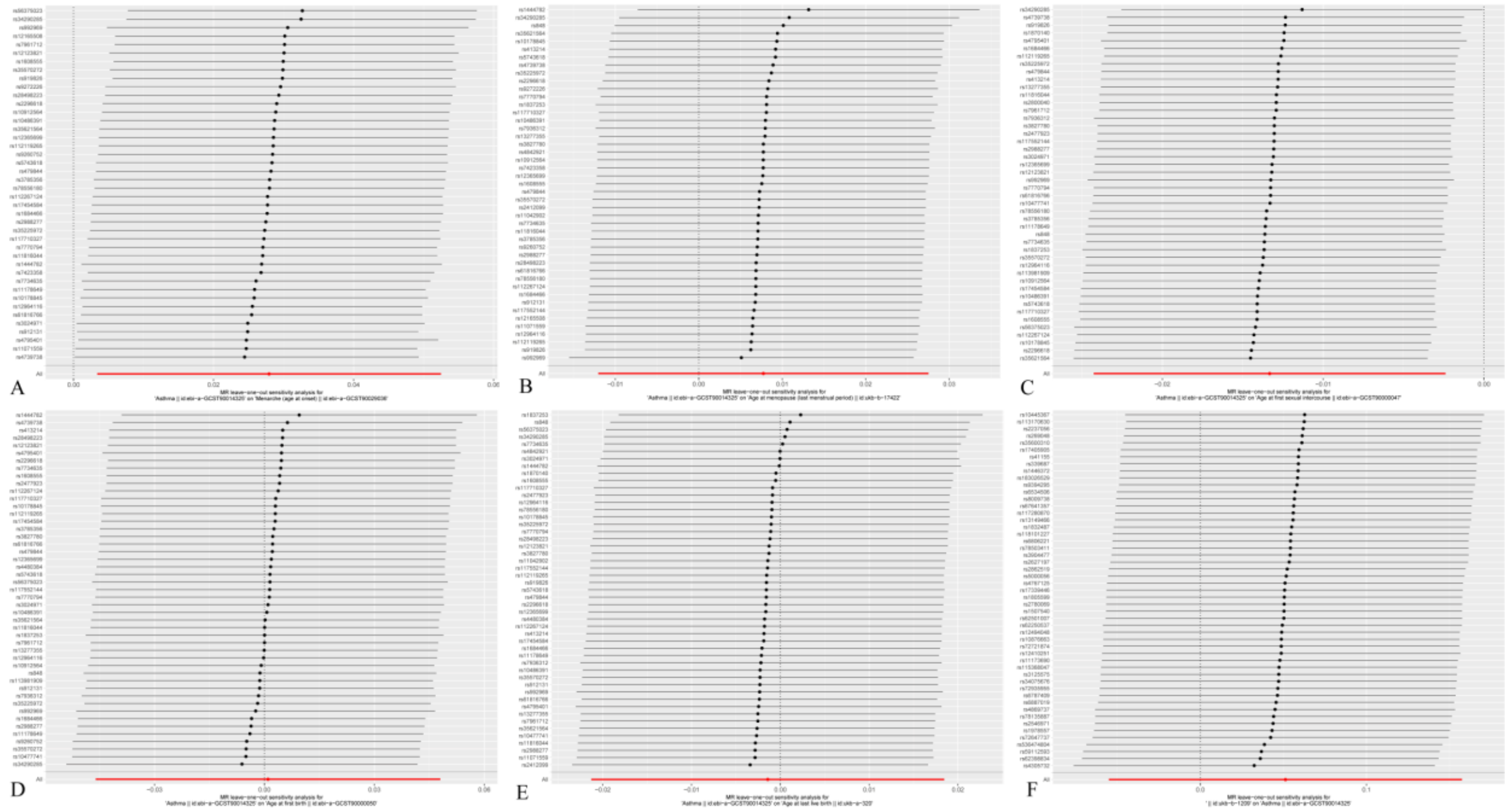

Supplementary Figure 10. The "leave-one-out" plots of the causality between asthma on women's reproductive traits**.** (A) Number of stillbirths; (B) Number of SM; (C) Hysterectomy; (D) Bilateral oophorectomy; (E) Ever used HRT; (F) Ever taken OC pill.

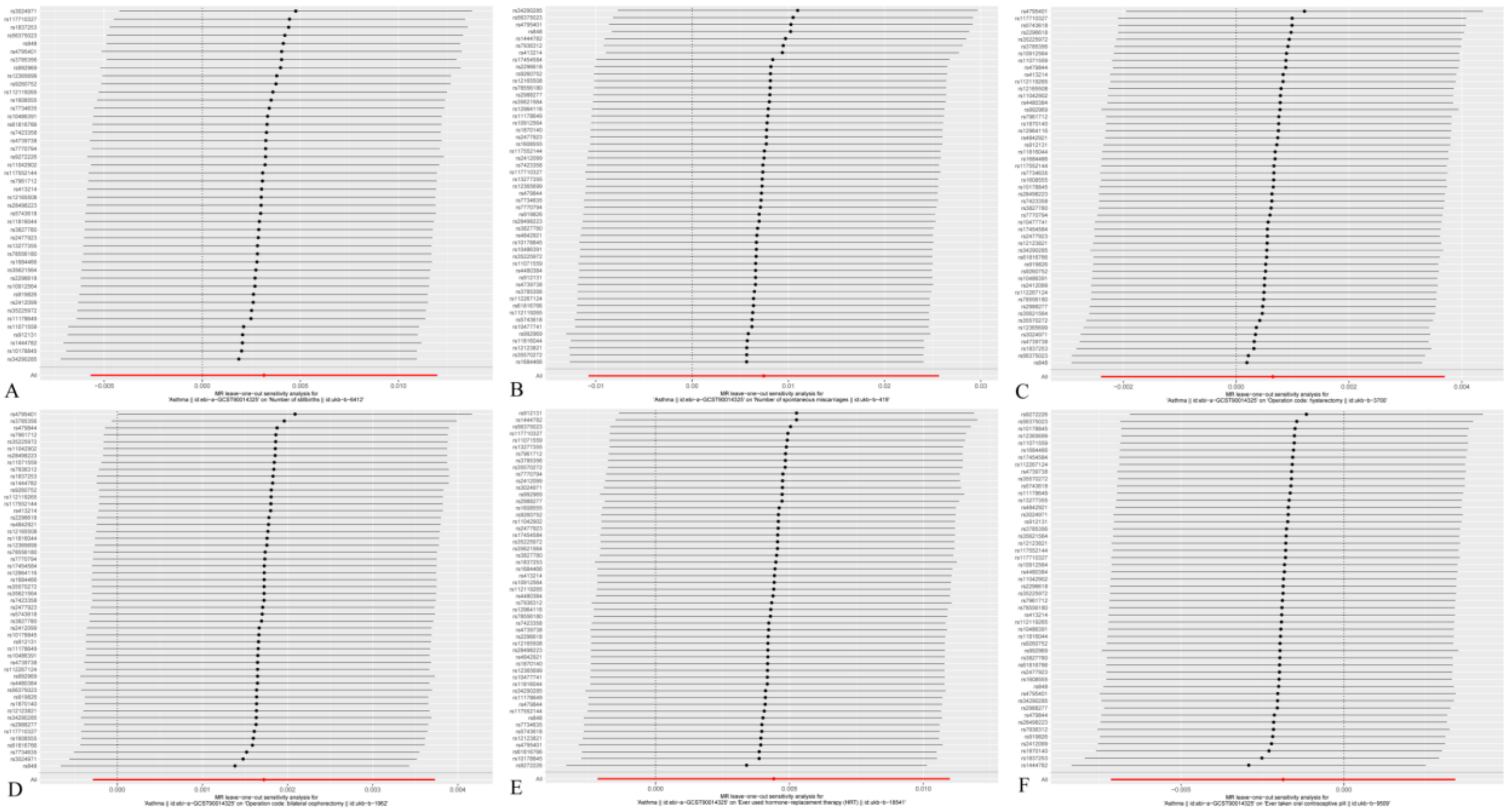

Supplementary Figure 11. The "leave-one-out" plots of the causality between pediatric asthma on women's reproductive traits**.** (A) AAM; (B) ANM; (C) AFS; (D) AFB; (E) ALB; (F) Number of live births.

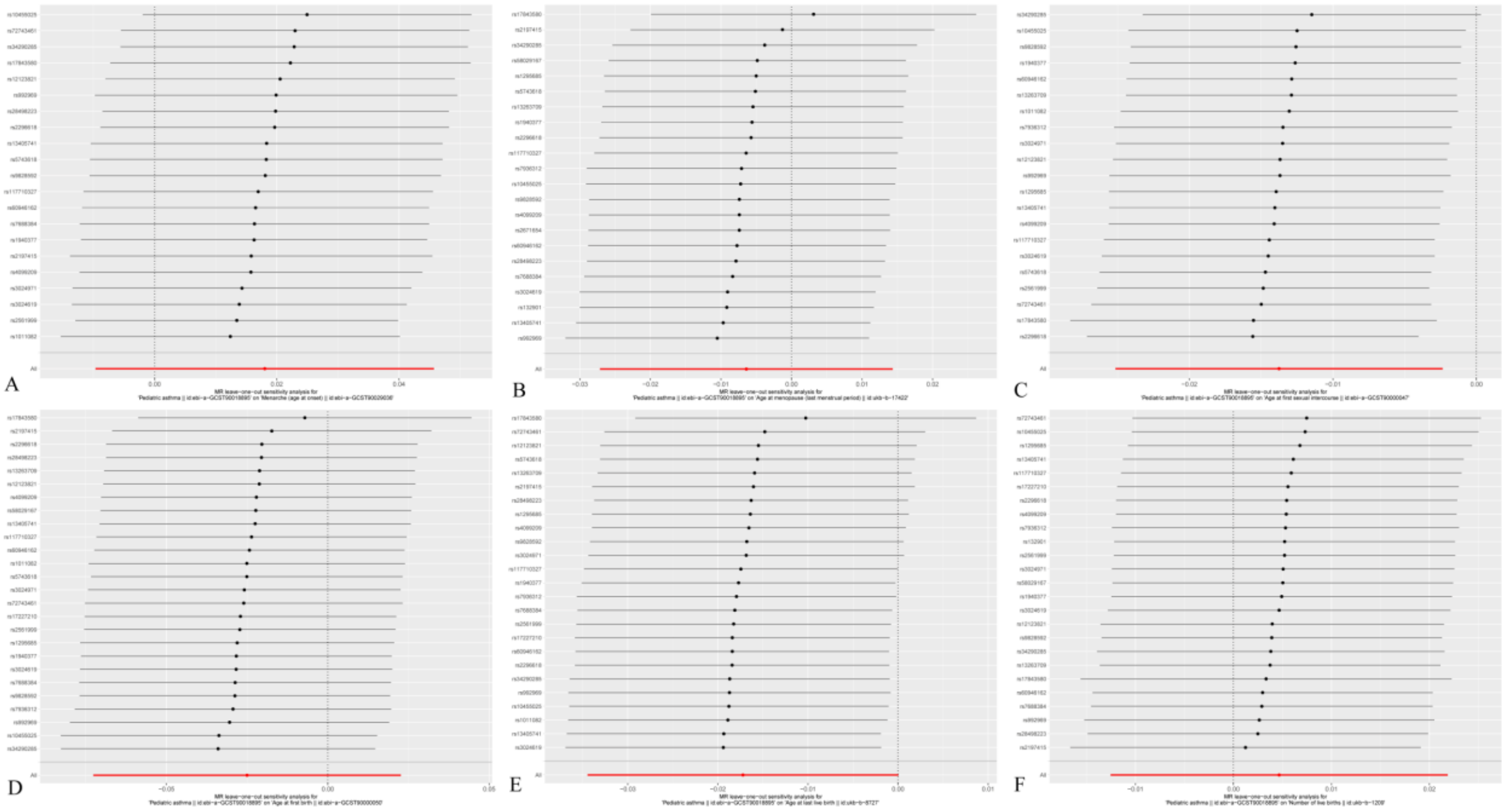

Supplementary Figure 12. The “leave-one-out” plots of the causality between pediatric asthma on women's reproductive traits**.** (A) Number of stillbirths; (B) Number of SM; (C) Hysterectomy; (D) Bilateral oophorectomy; (E) Ever used HRT; (F) Ever taken OC pill.

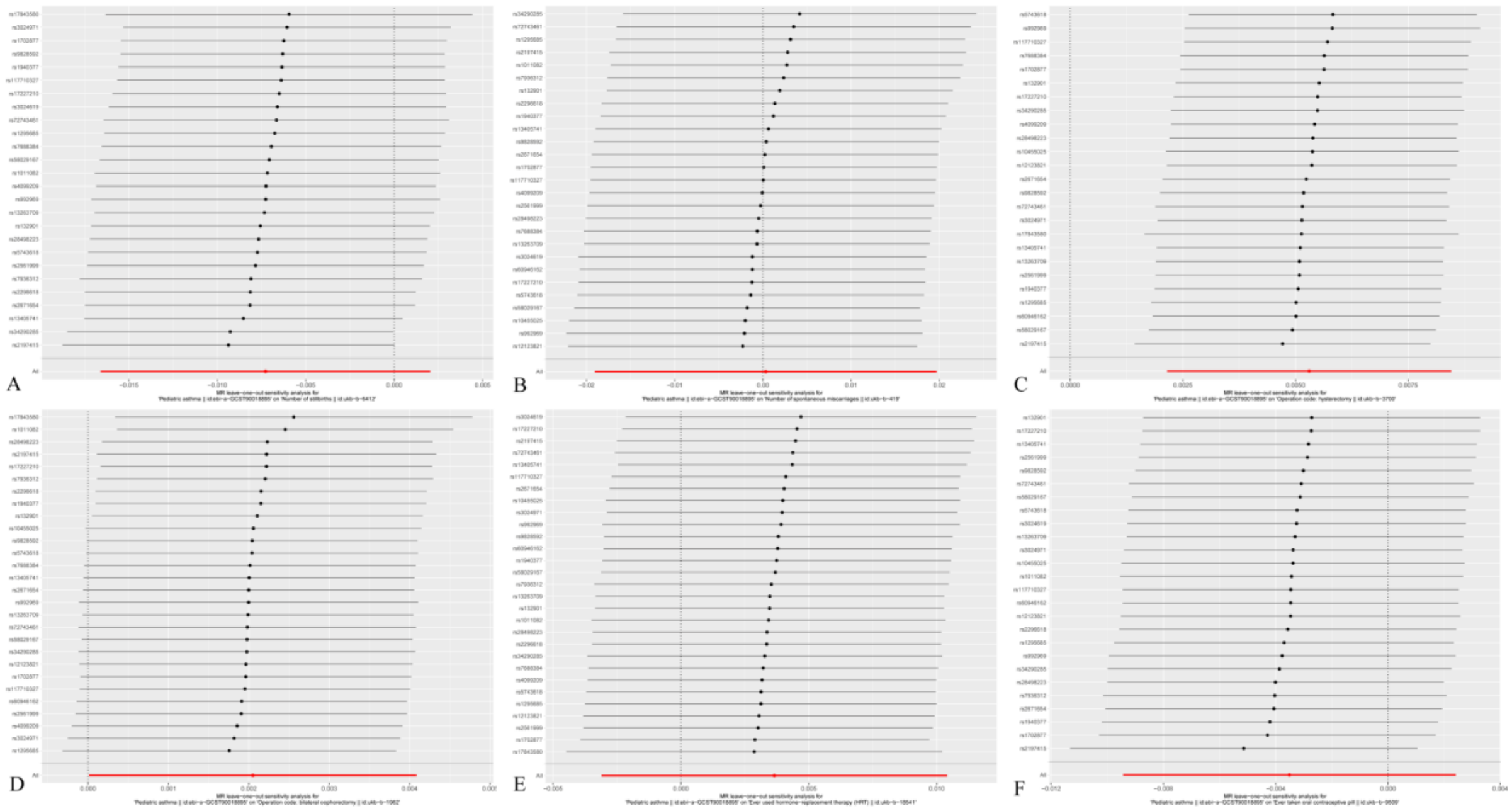

Supplement: Supplementary file 1 [file medi-104-e44720-s002.pdf]
